# Single-dose radiosurgical treatment for hepatic metastases - therapeutic outcome of 138 treated lesions from a single institution

**DOI:** 10.1186/1748-717X-8-175

**Published:** 2013-07-09

**Authors:** Daniel Habermehl, Klaus K Herfarth, Justo Lorenzo Bermejo, Holger Hof, Stefan Rieken, Sabine Kuhn, Thomas Welzel, Jürgen Debus, Stephanie E Combs

**Affiliations:** 1 Department of Radiation Oncology, University Hospital Center Heidelberg, INF 400, Heidelberg 69120, Germany; 2 Institute of Medical Biometry and Informatics, University Hospital Heidelberg, INF 305, Heidelberg 69120, Germany; 3 Klinik für Radioonkologie, University of Heidelberg, INF 400 69120, Heidelberg, Germany

## Abstract

**Background:**

Local ablative therapies such as stereotactically guided single-dose radiotherapy or helical intensity-modulated radiotherapy (tomotherapy) with high single-doses are successfully applied in many centers in patients with liver metastasis not suitable for surgical resection. This study presents results from more than 10 years of clinical experience and evaluates long-term outcome and efficacy of this therapeutic approach.

**Patients and methods:**

From 1997 to 2009 a total of 138 intrahepatic tumors of 90 patients were irradiated with single doses of 17 to 30 Gy (median dose 24 Gy). Median age of the patients was 64 years (range 31–89 years). Most frequent underlying tumor histologies were colorectal adenocarcinoma (70 lesions) and breast cancer (27 lesions). In 35 treatment sessions multiple targets were simultaneously irradiated (up to four lesions at once). Local progression-free (PFS) and overall survival (OS) after treatment were investigated using uni- and multiple survival regression models.

**Results:**

Median overall survival of all patients was 24.3 months. Local PFS was 87%, 70% and 59% after 6, 12 and 18 months, respectively. Median time to local progression was 25.5 months. Patients with a single lesion and no further metastases at time of RT had a favorable median PFS of 43.1 months according to the Kaplan-Meier estimator. The type of tumor showed a statistical significant influence on local PFS, with a better prognosis for breast cancer histology than for colorectal carcinoma in uni- and multiple regression analysis (p = 0.05). Multiple regression analysis revealed no influence of planning target volume (PTV), patient age and radiation dose on local PFS. Treatment was well tolerated with no severe adverse events.

**Conclusion:**

This study confirms safety of SBRT in liver lesions, with 6- and 12 months local control of 87% and 70%. The dataset represents the clinical situation in a large oncology setting, with many competing treatment options and heterogeneous patient characteristics.

## Introduction

Liver metastases frequently occur in patients with cancer, with the incidence varying depending on the underlying type of disease. The most frequent underlying tumor is localized in the gastrointestinal tract, predominantly colorectal cancer; in these patients approximately 25% of the patients present with liver metastases at primary diagnosis, and around 50% have been reported to develop liver lesions within 5 years from initial diagnosis [1]. In the past, radiation therapy played only a minor role in the treatment of liver metastases, due to the low tolerance of the normal liver tissue to radiation [2]. Radiation-induced side effects, namely RILD (Radiation Induced Liver Disease) were expected and thus RT was used only in rare cases. Therefore, surgery or other locally ablative alternatives were preferred in these patients.

In case of oligometastasis to the liver surgical resection is still the standard treatment, and locally ablative treatments are often held back and are applied in heavily pre-treated patients with multiple lesions to the liver. Considering the outcome of patients with liver metastases, outcome substantially depends on cancer histology: In patients with colorectal carcinoma survival rates of up to 64% five years after intervention can be achieved after surgical resection [3]. A recent metaanalysis on surgical resection of liver metastases derived from breast cancer describes a median overall survival of 40 months and a median 5-year survival rate of 40% in a selected patient cohort [4]. However, in a substantial number of patients, unfortunately, liver lesions are not amenable to resection, or patients suffer from severe comorbidities or present with reduced overall performance status; thus, surgical intervention may not be possible in these patients [5,6].

Locally ablative treatments such as radiofrequency ablation (RFA), percutaneous ethanol injection (PEI) or transcatheter arterial chemoembolization (TACE) are therefore widely accepted alternative treatment approaches with promising local control rates [7-9]. Nevertheless, minimal-invasive techniques still have major limitations such as tumor size and localization, and the individual tumor blood supply. In many cases, tumors still recur after a few months [10].

Modern radiation techniques have modified the role of radiation therapy in this clinical situation and have led to an establishment of this treatment modality in liver cancer patients. Thus, application of even high tumoricidal doses can be applied safely and non-invasively by stereotactic body radiotherapy (SBRT), intensity-modulated RT (IMRT) or even particle beam therapy (PBT) [11-21]. Many reports over the last years have shown favourable local control rates after radiosurgery or hypofractionated RT in liver metastases or even small primary liver tumors with an advantageous toxicity profile [22-29].

The present study reports long-term follow-up of a mono-institutional experience including 90 patients with 138 hepatic tumors treated at our institution.

## Patients and methods

### Patients and treatment

From 1997 to 2009 one hundred thirty-eight intrahepatic tumors from ninety patients were irradiated with total doses of 17 to 30 Gy (median dose 24 Gy) using stereotactic body radiotherapy (SBRT) or helical intensity-modulated radiotherapy (tomotherapy). Generally, following our institutional guidelines, these patients are treated with radiosurgical approaches. The decision on whether RT was performed by SBRT or HT (helical tomotherapy) was made according to current treatment capacities in our institution and had no medical or dosimetric reasons.

Median age was 64 years (range 31–89 years). A total of 126 lesions were irradiated using SBRT and 12 lesions using tomotherapy. Applied single fraction doses varied from 17 to 30 Gy and in most treatments the following doses were prescribed: 24 Gy (41 pts.), 20 Gy (37 pts.), 28 Gy (24 pts.) and 22 Gy (14 pts.) (Table 1).

**Table 1:** Patient and treatment details

**Total number of analyzed patients/lesions**	**90 patients/138 lesions**
Gender	
Male	47 patients, 75 lesions
Female	43 patients, 63 lesions
Age [y]	
Median, range [years]	64 (31 – 89)
Radiation technique [number of lesions]	
Single-dose (radiosurgery)	138
Stereotactic Body RT (SBRT)	126
Helical IMRT (tomotherapy)	12
Treatment in 1 session	
One lesion	62
Two lesions	28
Three lesions	6*
Four lesions	1
RT Dose	
Median, range [Gy]	24 (17 – 30)
Applied doses	Number
17–20 Gy	43
21–25 Gy	60
26–30 Gy	35
Previous treatment	Number of patients
Surgery	18
RFA	3
LITT	3
TACE	1
Other metastases at time of RT	Number
None	53
History of metastases	48
Simultaneous metastases	37
Planning Target Volume (median, range)	62 ml (11 – 333 ml)
Liver volume	1483 ml (range 982–2647 ml)

*1 patient with three lesions included in 1 target volume.

*RT* radiotherapy, *LM* liver metastasis, *RFA* radiofrequency ablation, *TACE* transcatheter arterial chemoembolization, *LITT* laser-induced thermotherapy.

In most cases dose was prescribed to the 80%-isodose in case of single-dose RT. In some cases the applied dose was prescribed to the dose maximum when appropriate, depending on the size of the lesion, proximity to organs at risk such as the intestines, or proximity to other radiation treatment volumes. In 28 cases two lesions were irradiated in one session, six patients received single-dose RT to a PTV encompassing three different lesions and in one patient four lesions were treated simultaneously. Six patients received more than one radiation course because of recurrent lesions during observation period. Median irradiated planning target volume was 62 ml (range 11–333 ml) and average normal liver volume was 1483 ml (range 982–2647 ml) (Table 1).

The most frequent primary tumor site was colorectal adenocarcinoma (70 pts.), followed by breast cancer (27 pts.), pancreatic adenocarcinoma (11 pts.) and ovarian cancer (7 pts.) (Table 2).

**Table 2:** Primary tumor sites

**Primary tumor site**	**Number of treated liver metastases**
**TOTAL**	**138**
Colorectal adenocarcinoma	70
Adenocarcinoma of the breast	27
Pancreatic adenocarcinoma	11
Ovarian cancer	7
Lung cancer	6
Gastric cancer	3
Sarcoma	2
Cholangiocarcinoma	2
Esophagus	2
Endometrium	2
Lymphoma	1
Renal Cell Cancer	1
Cervival cancer	1
Adenoid-cystic carcinoma	1
Anal Cancer	1

### Patient immobilization and treatment planning

Patients were immobilized as described previously [22,30]. In brief, patients were immobilized using an individually shaped vacuum pillow and an abdominal compression to reduce the liver movement. A contrast agent enhanced CT scan and a 4D-CT series for quantifying liver motion was acquired for treatment planning.

The extracranial stereotactic set-up has been developed at the German cancer research center (dkfz) and is commercially available (Leibinger, Freiburg, Germany). The patient is positioned in an individually shaped vacuum pillow (Brandis Medizintechnik, Weinheim, Germany). The intra-corporal movement of the liver was reduced by epi-gastrical compression using a triangular Plexiglas plate. Fixation of the plate is performed by two bars, which are firmly attached to the metal arch. A Siemens somatom plus 4 (Siemens, Erlangen, Germany) was used for treatment planning. A spiral CT scan with 5 mm slice thickness and 500 mm field of view was performed which included the localization system. The patients were advised to breathe normally during the scanning time without taking deep breaths.

On the treatment day patients were repositioned in the above mentioned setting using pen marks and another control CT scan. Until the year 2003, CT imaging was performed offline, and, if positioning was adequate, patients were brought to the linear accelerator (LINAC) using an individual shuttle system leaving the patient in the vacuum bag/abdominal press fixation. In recent years (2004–2009), LINACs (e.g. Siemens Artiste, Siemens Healthcare, Erlangen, Germany) were equipped with on-board imaging as, e.g. in case of tomotherapy, a combination of the 6 MeV LINAC with CT imaging.

Target and OAR contouring was performed using Siemens Dosimetrist and Oncologist software, and inverse treatment planning was conducted applying the Hi-ART Tomotherapy planning software (TomoTherapy Inc., Madison, WI, USA). Dose constraints for adjacent organs at risk and the liver were used accroding to Emami et al. and Dawson et al. [31,32].

### Follow-up

Median follow-up was 21.7 months (range 1.6 – 151.8 months). Local failure patterns were determined by follow-up clinical examination, radiographic imaging including CT- and MRI scans as well as ultrasound. Overall survival (OS) was calculated from the treatment day on. Local Progression-free survival was determined as the period between the treatment day and appearance of any local recurrence; data was censored in case of death without progression or last follow-up in patients without progression. Univariate survival curves were plotted based on the Kaplan-Meier method. In addition to univariate analyses, multiple regression relying on a proportional hazard (Cox) model was performed. Statistical calculations were implemented using SAS version 9.2 (SAS Institute, Cary, NC, USA), and survival curves were plotted using R 2.11.0 (http://www.rproject.org).

## Results

### Overall survival (OS)

**Figure 1:**
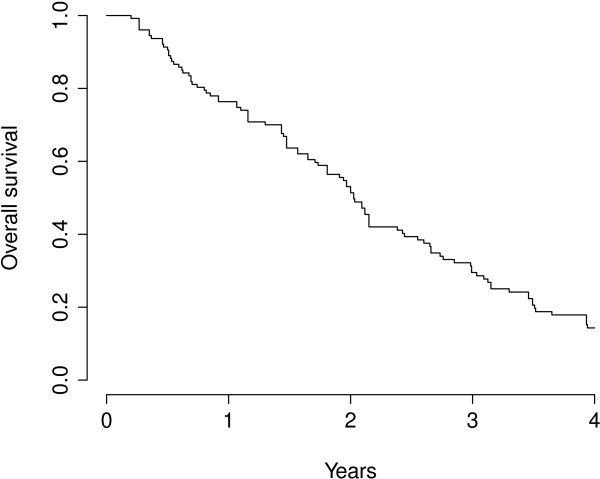
Kaplan-Meier overall survival curve for all patients.

The median OS for all patients was 24.3 months (95%-CI 20.8 – 28.6 months), the corresponding survival curves are represented in Figure 1. Relevant factors associated with a poor prognosis according to univariate analyses were the primary site of the tumor (HR = 2.56 for ‘other’ compared to colorectal tumors, global p = 0.002), the number of lesions (p < 0.001) and a late calendar year (2001–2009) at diagnosis (HR = 1.52, p = 0.04). Multiple regression survival analysis consistently pointed out to a poorer prognosis of patients with metastases outside the liver at the time of RT or a history of metastasis (HR = 2.64, p = 0.003), and also the number of simultaneously irradiated lesions showed an effect on OS (p = 0.02). Patients with a primary tumor histology of colorectal cancer had a better prognosis than other histologies (HR = 4.39, p = 0.0009).

### Progression-free survival

**Figure 2:**
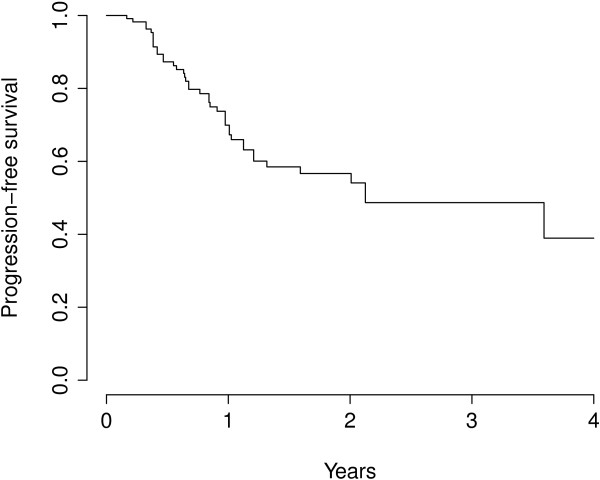
Local progression-free time (PFS) of lesions.

**Figure 3:**
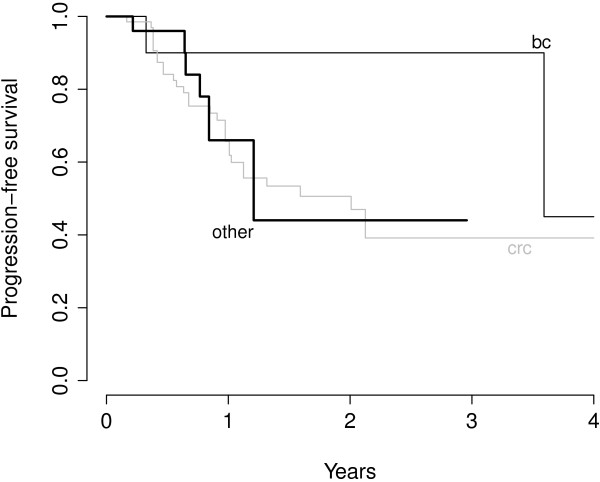
**Local progression-free survival of patients with metastases from colorectal carcinoma (CRC) and adenocarcinoma of the breast (BC).** Kaplan-Meier curve of PFS according to primary tumor site.

The proportions of lesions without local progression after 6, 12 and 18 months were 87.3%, 69.9% and 58.5% respectively. The median time to local progression was 25.5 months for the entire patient group (Figure 2). The number of metastases (one vs. multiple) that were included in one or more PTVs or were irradiated simultaneously during one session showed no influence on local control (p = 0.76). The type of tumor showed an influence on the local control of irradiated metastases, lesions from patients suffering from colorectal carcinoma (‘crc’) showed a poorer control than those from patients affected by breast cancer (‘bc’) in univariate and multiple regression analyses (multivariate HR for breast cancer 0.15, p = 0.05). The survival curve on Figure 3 suggest that, after single-dose SBRT, lesions in patients with metastasis from breast cancer show a slower local progression.

The effects on progression of planning target volume (PTV) (11–59 ml or 60–333 ml), patients’ age (31–59 or 60–89 years), primary tumor site (colorectal cancer, breast cancer, other), number of metastases (singular vs. multiple) and applied radiation dose (17–22 Gy, 23–24 Gy, >25 Gy) did not reach statistical significance according to multiple Cox regression. Patients with a single lesion had a median PFS of 43.1 months according to the Kaplan-Meier estimator, but survival differences between patients with single lesions and those patients with other lesions than the treated ones at time of RT did not reach statistical significance according to uni- and multiple regression models (univariate: p = 0.32, multivariate: p = 0.41).

### Toxicity

Overall tolerance of RT was high. Radiation side effects were mild and included in some cases mild fever, nausea, chills, loss of appetite and transient elevation of alkaline phosphatase. No Radiation-induced-liver disease (RILD) was observed in the entire patient group.

## Discussion

In the present work we report our long-term follow up of highly conformal radiation therapy in patients with liver metastases delivered as radiosurgery. In this cohort of 90 patients with 138 metastatic lesions, local control was nearly 90% after six months. The main underlying tumor type was colorectal cancer, followed by breast cancer. Overall survival in the whole group was 24.3 months, and prognostic factors included primary tumor histology. In our analysis of this patient group with a wide variety of different underlying primary tumors and different systemic and local pre-treatments, local control rates within the range of published data (Table 3) can be achieved with single fraction RT even in case of multiple target lesions.

**Table 3:** Literature overview

**Author**	**Year**	**Primary tumour**	**Number of patients**	**Number of fractions**	**Overall dose**	**Local control**	**Details**
Herfarth et al. [14]	2001	Mets and PLC	56	1	14 – 26 Gy	67% after 18 months	
Wulf et al. [15]	2001	Mets and PLC	56	3	27 pts. 3 x 10 Gy 1 pt. 4 x 7 Gy	61% after 2 years PLT: 100% after median f/u 15 mo.	
					19 pts. 3 x 12-12.5 Gy		
					1 pt. 1 x 26 Gy		
Schefter et al. [16]	2005	Mets	18	3	9 Pts.: 36 Gy	Not reported	Phase-I study
					9 Pts.: 60 Gy		
Hoyer et al. [17]	2006	Mets	44	3	45	Not reported	Phase-II study, Liver-specific data not reported
Kavanagh et al. [18]	2006	Mets	36	3	60 Gy	93% after 18 months	Phase-I/II study
Mendez-Romero et al. [19]	2006	Mets and PLC	45	3	37.5 Gy	82% after 2 years	Phase-I/II study
					(also 5 x 5 Gy or 3 x 10 Gy)		
Katz et al. [27]	2007	Mets	174	7 – 20	30 – 55 Gy	57% after 20 months	Lesion diameter ranged from 0.6 – 12.2 cm
					median 48 Gy		
Rusthoven et al. [11]	2009	Mets	63	3	36 – 60 Gy	95%, 92% after 1 and 2 years	Phase-I/II study
Lee et al. [12]	2009	Mets	68	6	27.7 – 60 Gy	71% after 1 year	Phase-I study
					median 41.8 Gy		
Van der Pool et al. [20]	2010	Mets	31	3	12.5 Gy (n = 29)	74% after 2 years	Grade III toxicities were hepatic
					15 Gy (n = 2)		
Goodman et al. [21]	2010	Mets and PLC	40	1	18 – 30 Gy	77% after 1 year	Phase-I study
Rule et al. [28]	2011	Mets	37	3 or 5	30 – 60 Gy	56%, 89%,100% for 30, 50 and 60-Gy after 2 years	Phase-I study
					(30 Gy/3 fx, 50 and 60 Gy in 5 fx)		
Chang et al. [29]	2011	Mets	65	1-6	22–60 Gy, median 42 Gy	67%, 55% after 1 and 2 years	
Vautravers-Dewas et al. [41]	2011	Mets	42	3-4	40 Gy/4 fx, 45 Gy/3 fx	86% after 2 years	
Lanciano et al. [40]	2012	Mets and PLC	30	3 or 5	79.2–180 Gy10	75%, 57% with BED10 >100 Gy after 1 and 2 years	
					(66–150 Gy BED2)		
**This study**	**2013**	**Mets**	**138**	**1**	**10-30 Gy, median 24 Gy**	**87%, 69% and 59% after 6, 12 and 18 months**	

HCC without cirrhosis, and HCC < 4 cm with cirrhosis were mostly treated with 3 ×12.5 Gy. Patients with HCC ≥ 4 cm and cirrhosis received 5 × 5 Gy or 3 × 10 Gy. Gy Gray, CRC colorectal carcinoma, mets metastases, Pts. Patients, Pt. patient, DLT Dose-limiting toxicity, PLC Primary Liver cancer, fx fractions, LC Local Control.

In general, the development of SBRT or other techniques to deliver precisely high local doses have changed the paradigm for radiation therapy in the multimodal treatment of liver metastases. Radiation tolerance of liver normal tissue is limited in contrast to the high local doses required for long-term local control of metastatic lesions. The dose-limiting toxicity of the whole liver is RILD, which in the early days was named radiation hepatitis [33]. It is characterized by a rise in liver enzymes, namely alkaline phosphatase, and ascites; histopathologically, venous occlusion is the most predominant change in pathological specimens [34].

Respecting individual target volumes and dosing schemes, toxicity rates can be minimized, even for larger volumes. In contrast to primary liver cancer, liver metastases most commonly occur in non-cirrhotic livers, and the most predominant toxicity to radiation will be RILD; however, adhering to established dose constraints of normal liver tissue, i.e. keeping below 30 Gy median liver dose in conventional fractionation [31] or application of less than 15 Gy to 700 ml of healthy liver tissue, this may be securely avoided. Most of the studies report mild adverse events such as transient elevation of alkaline phosphatase or fatigue symptoms, toxicity grade III or higher (CTCAE) is very uncommon and occurs only when high doses are applied to the small bowel or to large volumes of the liver [35]. However in our analysis no higher grade toxicities were recorded, especially no clinically apparent RILD. Nevertheless a major weakness of this work remains the documentation of detailled toxicity aspects according to the Common Toxicity Criteria for Adverse Events (CTCAE) because of the retrospective nature of the study. The available medical records and documents did not point out to higher grade of clinically relevant adverse events, e. g. gastro-intestinal ulceration. Therefore we refer to published reports on comparable radiosurgery treatment protocols where mostly mild toxicities are observed and higher grad toxicities are surely very rare events (see Table 3).

Median overall survival of patients with untreated liver metastasis differs among histologies of the primary tumor between 5 and 19 months [36,37]. After surgical resection a five-year OS of 64% can be achieved [3]. Patients should only undergo resection if clear resection margins can be achieved (R0-resection), because positive margins (including R1- and R2-resections) are associated with a poor five-year OS of 0% in many case series [38,39]. While surgery is the mainstay of treatments, substantial numbers of patients are not amenable for surgical resection, often for underlying medical conditions [5,6]. Therefore a current need for local ablative treatment options in liver metastasis is evident. With the advancing techniques of SBRT and other elaborate modalities allowing for precise local high dose deposition, several institutions have treated a large number of patients with liver metastases, either applying radiosurgery or hypofractionated regimens (Table 3). Survival of patients in our analysis is relatively good and in the range of the above mentioned group of patients undergoing surgery. Median OS was 24.3 months and depending on the prognostic subgroup (breast cancer histology, no further metastases at time of RT) median local PFS is even higher.

Recently a phase-I clinical trial examining a dose escalation with single-fraction SBRT from initially 18 to 30 Gy in 19 patients with hepatic metastases has shown efficacy without reporting dose-limiting toxicity [21]. Risk for local failure 12 months after treatment was 23%. Table 3 provides a summary of the largest studies on SBRT of liver metastases, including single-fraction RT as well as the more commonly used 3–5 fraction protocols. Overall, local control ranges between 70 and 95% at one year, depending on the series; however, only few studies have been conducted using a formal dose-escalation part. Rusthoven and colleagues performed a multicentric phase I/II clinical trial starting with 36 Gy at 3 fractions, and dose was escalated in 6 Gy increments up to a defined maximum of 60 Gy [11]. Tumor volume was a strong predictor for local control, with smaller lesions showing a longer tumor control. Colleagues from the University of Texas Southwestern reported on a Phase I study starting with 30 Gy in 3 fractions up to 60 Gy in 5 fractions [28]. An argument for the five-fraction scheme was the potential to treat lesions in close vicinity to periportal biliary structures avoiding radiation-induced toxicity with lower single doses. A third dose-escalation study was published by Goodman et al. and was phase I dose-escalation single-fraction trial including not only patients with liver metastases, but also with intrahepatic cholangiocarcinoma [21]. Dose escalation was conducted in 4 Gy treatment groups from 18–30 Gy, in a single fraction. Also in this study, no dose limiting side effects were observed. A recent mono-institutional analysis of heavily local and systemic pre-treated patients with liver tumors undergoing cyber knife ®-based RT showed the feasibility and efficacy of 3–5 fraction regimens with doses of BED ≥ 79.2 Gy10 = 66 Gy EQD2 [40]. In this setup remarkable LC rates of up to 74% after 2 years can achieved with doses of BED ≥ 100 Gy10. But there are also some reports indicating higher local control rates of approximately 90% and higher for CRC patients after 3-fraction SBRT, when doses of 45–48 Gy (BED) are applied [29,41].

As a summary of available studies it be may be concluded that SBRT for liver metastasis leads to a 1- and 2-year local control of approximately 60-100% depending on patient group and fractionation scheme (recently reviewed by [35]). With regard to these data, our data reported in the present manuscript fit well in the range of reported data, confirming 12- and 18-months PFS rates of 70% and 59%; however, in our group, patients presented with heterogeneous primary tumors, pretreatments, comorbidities and lesion characteristics. In comparison to many prospective studies, this dataset represents more a “real-life” scenario reflecting the clinical situation in a large oncology setting, with many competing treatment options. Additionally, the data confirms safety of the method in this group of patients, also for larger lesions in heavily pretreated patients. However, while reporting these real-life data, limitations of such an analysis must be kept in mind, such as some patients being lost-to-follow-up, or some data limitations due to the retrospective nature of the report.

The most analysed patient group of SBRT treated liver metastasis are patients with metastases derived from colorectal cancer. In several studies outcome of this patient group was relatively worse compared to other primary tumors which also can be confirmed by our analysis with 1-year local control rates of 67% for CRC compared to 90% for breast cancer patients [12,42]. One possible explanation of this observation may be the fact that CRC patients are often heavily pre-treated with several chemotherapy regimens and may therefore develop a cross resistance to ionizing radiation; moreover, due to the prior chemotherapeutic regimens patients are referred at a later stage of their disease, which might also explain the reduced outcome in CRC patients.

With increasing lesion volume safety concern with single fraction treatments become evident. Thus, multi-fraction schedules using 3–6 fractions may provide a better risk-benefit profile. However median lesion size of irradiated metastases certainly varies among different studies. While some authors report tumor size with the longest diameter or volume, others analysed the planning target volume, as we did in our study. Notably some authors found a dose-volume relationship [11,12] whereas our analysis failed to support a dependency of PTV and tumor response. Recent reports of other groups also failed to show a dependency between size and LC [29,40,41].

Future studies in this field will elucidate the benefit of more (tumor-) adaptive radiotherapeutic procedures. Using highly conformal image-guided radiotherapy intrahepatic tumors can be irradiated with smaller safety margins, thus normal liver tissue can be prevented from radiation damage. For this purpose, e.g. fiducial markers are implanted in proximity of the target lesion and can be visualized by computed tomography or x-ray images as a tumor surrogate to assure its correct position during treatment. New technologies such as gating and tracking of target lesions depend on the breathing phase and are correlated with the dose application [43-45].

Finally many retrospective studies and phase-I/-II clinical trials on SBRT have shown a high local control rate of intrahepatic metastases while avoiding severe therapy-related complications in patients not suitable for resection. Best results were achieved with hypofractionated regimens and overall dose above 40 Gy in selected patient groups. The role of radiation therapy for the treatment of patients with liver metastases should therefore be re-defined and kept in mind in interdisciplinary treatment decisions. Further clinical evaluation, preferentially in randomized settings comparing to surgery of other locally ablative techniques, will further elucidate the full potential of SBRT in patients with liver metastases, especially in the subgroup of oligometastasized patients. Nevertheless prospective randomized clinical trials (RCT) comparing different local ablative therapies, e.g. TACE/RFA and SBRT, are still lacking. According to this issue most recently Hoyer et al. initiated a multi-institutional RCT comparing RFA and SBRT for hepatic oligometastasis (1–4) (RAS01-Trial, NCT01233544) [35].

## Conclusion

Our analysis of a large retrospectively evaluated mono-institutional patient group who was treated with single-dose SBRT for liver metastases clearly shows a good local control rate. Many of these patients had more than one intrahepatic lesion or suffered from multiple metastases during treatment. Moreover there were different underlying primary tumors with impact on local control. In summary the analysis provides further evidence of the efficacy of highly conformal radiation, serving a basis for additionally radiotherapeutic improvements or a rationale for randomized trials with other locally ablative treatments or surgery. The dataset supports the use of SBRT in liver lesions, also in heavily pretreated patients or with larger lesions, with a convincing safety profile and sufficient lesion control probability.

## Competing interests

The authors declare that they have no competing interests.

## Authors’ contributions

KH, HH, TW, JD, SK and SEC were responsible for patient treatment and care. KH, HH, SK and JD established the SRS methods. DH and KH collected the patients’ data. JLB performed all statistical analyses. DH collected the data and wrote the manuscript. DH, KH, SR, TW, HH, JD and SEC contributed to the analysis of data and revised the manuscript. SEC conceived the study, helped to write and finalized the manuscript. All authors helped with the interpretation of the data, read and approved the final manuscript.
